# The Effect of Joint Mobilization and Manipulation on Proprioception: Systematic Review with Limited Meta-Analysis

**DOI:** 10.3390/jfmk11010059

**Published:** 2026-01-29

**Authors:** Stelios Hadjisavvas, Irene-Chrysovalanto Themistocleous, Michalis A. Efstathiou, Elena Papamichael, Christina Michailidou, Manos Stefanakis

**Affiliations:** Department of Health Sciences, Physiotherapy Program, University of Nicosia, Nicosia 1700, Cyprus

**Keywords:** spinal manipulation, peripheral joint mobilization, sensorimotor control, joint position sense error, joint repositioning error

## Abstract

**Background:** Proprioceptive deficits, commonly quantified as joint position sense error (JPSE), are frequently reported in musculoskeletal conditions. Articular manual therapy may influence afferent input and sensorimotor integration. This review synthesised the effects of joint mobilization and/or high-velocity low-amplitude (HVLA) thrust manipulation on quantitative proprioception outcomes in humans. **Methods:** PubMed, Scopus, CINAHL, and MEDLINE Complete were searched (from inception to November 2025) for randomized or sham-controlled trials assessing proprioception after eligible articular manual therapy. Searches were limited to English-language publications. Risk of bias was assessed using Risk of Bias 2 (RoB 2). Random-effects meta-analysis (Hedges’ g) was conducted when outcomes and time points were comparable; pooling was possible for only one outcome/time-point comparison. Certainty of evidence was assessed using GRADE. **Results:** Database searches yielded 483 records; after duplicate removal, 371 records were screened. Eighteen full-text articles were assessed for eligibility, of which 11 were excluded, resulting in seven randomized clinical trials (2018–2025; total *n* = 350) evaluating spinal or peripheral mobilization/manipulation. No eligible randomized or sham-controlled trials meeting the prespecified criteria were identified before 2018. In chronic mechanical neck pain, cervical thrust manipulation improved cervical JPSE versus sham with large partial eta-squared effects (η^2^p = 0.23–0.36). Cervical mobilization improved left rotation JPSE (4.15 → 1.65° vs. 4.01→3.74°). In patellofemoral pain, lumbopelvic manipulation produced immediate reductions in knee JPSE at 60° (6.58 → 4.48° vs. 5.91 → 6.05°). Only one outcome/time-point was suitable for meta-analysis (knee JPSE at 60° flexion in patellofemoral pain; two trials), showing no statistically significant pooled effect (Hedges’ g = −0.21, 95% CI −1.36 to 0.94; I^2^ ≈ 83%). Remaining outcomes could not be pooled due to heterogeneity and incompatible reporting. **Conclusions:** Evidence from seven randomized trials indicates that articular manual therapy (mobilization and/or HVLA thrust manipulation) can improve quantitative proprioceptive outcomes immediately post-intervention, particularly JPSE in neck and patellofemoral pain; however, effects are condition- and outcome-specific, and confidence is limited by heterogeneity and the predominance of narrative synthesis with sparse poolable data. Future adequately powered trials should standardize proprioception protocols, include longer follow-up, and report data to enable robust meta-analysis.

## 1. Introduction

Musculoskeletal (MSK) conditions affect approximately 1.71 billion people worldwide and are among the leading contributors to disability globally, with low back pain being the single leading cause of disability in many countries [1]. In 2020 alone, low back pain affected an estimated 619 million people worldwide [2], while neck pain affected approximately 203 million people and remains a substantial contributor to years lived with disability (YLDs) [3].

These conditions commonly present in clinical practice as spine and peripheral joint pain with functional limitations, recurrent episodes, and reduced participation. Given their high disability burden, MSK disorders account for the largest global need for rehabilitation services, underscoring the importance of interventions that target both symptoms and underlying movement-related impairments [4].

Beyond pain intensity, many MSK presentations are characterised by altered sensorimotor function, including impaired proprioception, which may contribute to movement dysfunction and susceptibility to recurrent symptoms [5].

Proprioception denotes the awareness of body and limb positioning, movement, and force, derived from afferent signals from muscle spindles, Golgi tendon organs, joint receptors, and cutaneous mechanoreceptors, and processed at both spinal and supraspinal levels [6,7]. It is typically characterized by joint position sense (JPS), kinesthesia, and the perception of force or effort [6]. In this review, proprioception is used as the umbrella term for position, movement, and force sensing. Joint position sense (JPS) refers to the ability to reproduce a target joint position, whereas joint position sense error (JPSE) is the quantitative error metric derived from JPS tasks (lower values indicate better performance). For clarity, we use ‘quantitative proprioceptive outcomes’ or ‘proprioceptive performance’ when referring to measured endpoints such as JPSE. Precise proprioceptive processing is crucial for joint stability, postural control, and coordinated movement; conversely, deficiencies have been linked to discomfort, disrupted motor control, and heightened susceptibility to musculoskeletal damage [7,8].

Proprioceptive deficits have been documented in many musculoskeletal disorders. Elevated joint position sense error (JPSE) has been observed in individuals with neck discomfort, chronic ankle instability, and low back pain, signifying reduced proprioceptive performance in both spinal and peripheral joints [9,10,11]. Systematic reviews indicate that therapies aimed at proprioception or sensorimotor control may enhance proprioceptive performance and clinical outcomes in populations such as those with chronic neck pain, knee osteoarthritis, and ankle instability [12,13,14].

Manual therapy is extensively employed in the management of MSK disorders and commonly includes joint mobilization and high-velocity low-amplitude (HVLA) thrust manipulation [15]. In addition to mechanical impacts, modern models suggest that manual therapy may influence neurophysiological responses by modulating peripheral afferent input and sensorimotor integration [15,16]. Proprioceptive information is sent via mechanoreceptors that are mechanically activated during manual therapy; thus, these interventions are posited to affect quantitative proprioceptive outcomes (e.g., JPS/JPSE) [17].

Mechanistically, the transient mechanical stimulus delivered during joint mobilization or HVLA thrust manipulation may evoke a burst of afferent discharge from muscle spindles, Golgi tendon organs, joint receptors, and cutaneous mechanoreceptors, thereby altering the inflow of somatosensory information to the spinal cord and supraspinal centres [18,19,20]. Contemporary models propose that this sensory barrage can modulate segmental processing (e.g., reflex excitability and nociceptive transmission) and engage supraspinal pain- and sensorimotor-related circuitry, including descending inhibitory pathways [18,19,21]. At a neurochemical level, experimental work suggests that manual therapy-induced hypoalgesia may be mediated—at least in part—by monoaminergic mechanisms (e.g., serotonergic and noradrenergic pathways) and other neuromodulators implicated in descending pain modulation (e.g., endogenous opioids and endocannabinoids), although much of this evidence derives from preclinical models [21,22]. In addition, animal studies indicate that manipulation may influence inflammatory signalling (e.g., increased spinal anti-inflammatory cytokine IL-10), providing a plausible molecular pathway through which neuroimmune interactions could contribute to clinical effects [23].

Empirical evidence regarding the effects of manual therapy on proprioception remains inconsistent. Some trials have reported improvements in joint position sense (JPS) following mobilization or manipulation [24,25], whereas others have found no significant between-group differences despite changes in pain intensity or range of motion [26,27]. This variability may reflect differences in intervention characteristics (e.g., technique type, anatomical region, dosage, and single-session versus repeated exposure), participant profiles (symptomatic vs. asymptomatic), and outcome assessment (e.g., task, joint angle, and timing of measurement), all of which may influence proprioceptive responses [28].

Current evidence syntheses of manual therapy have predominantly prioritised clinical outcomes such as pain, disability, and function, whereas quantitative proprioceptive outcomes are seldom synthesised as primary endpoints [29,30,31,32]. In parallel, systematic reviews focused on proprioception and sensorimotor control largely emphasise exercise-based interventions, and when manual therapy is included, it is commonly embedded within multimodal programmes, limiting attribution of effects to the articular technique itself [12,13,14]. As a result, it remains unclear whether articular manual therapy alone (joint mobilization and/or HVLA thrust manipulation) produces measurable changes in quantitative proprioception (e.g., JPS/JPSE) and under which conditions such effects are most likely to occur.

This systematic review and meta-analysis was conducted to synthesise evidence from randomized or sham-controlled trials on the effects of articular manual therapy (joint mobilization and/or high-velocity low-amplitude [HVLA] thrust manipulation) on direct, quantitative proprioception outcomes (e.g., JPS and JPSE) in humans, compared with sham/placebo, no-intervention, or other control conditions.

Accordingly, the review was guided by the following questions:

(1) In humans, does articular manual therapy (mobilization and/or HVLA thrust manipulation) improve quantitative proprioception outcomes (e.g., JPS/JPSE) compared with sham/placebo, no intervention, or control conditions?
(2) Are effects dependent on the type of technique (mobilization vs. HVLA), anatomical region (spine vs. peripheral joints), participant status (symptomatic vs. asymptomatic), and timing of outcome assessment (immediate vs. follow-up)?
(3) Where data are sufficiently comparable, what is the pooled magnitude of effect on quantitative proprioception outcomes?

## 2. Materials and Methods

### 2.1. Study Design

**Figure 1:**
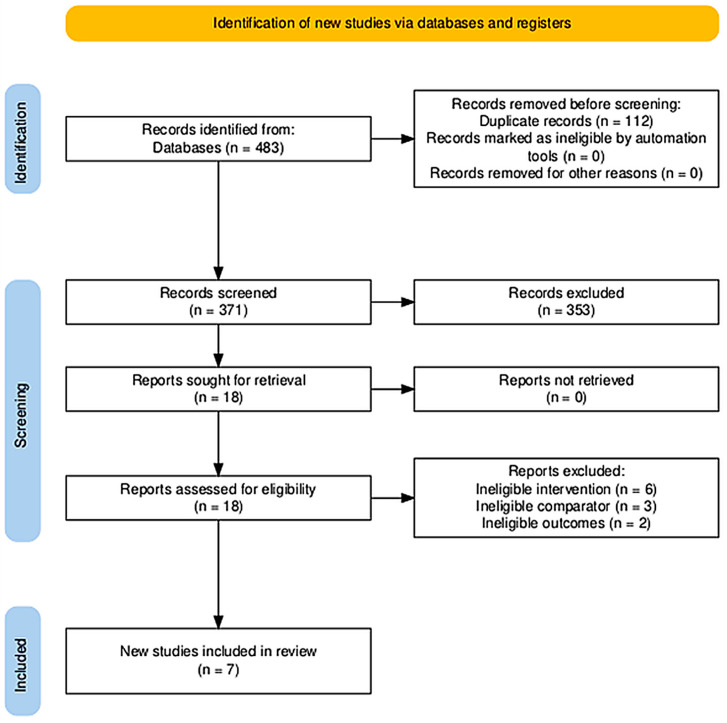
PRISMA flow diagram illustrating the process of study identification, screening, eligibility assessment, and inclusion in the systematic review.

This systematic review followed PRISMA 2020 [33] and was prospectively registered in PROSPERO (CRD420251161367). The PRISMA 2020 checklist is provided as Supplementary Table S1 and the PRISMA flow diagram as Figure 1. No protocol amendments were made after registration.

### 2.2. Eligibility Criteria

The eligibility criteria were defined utilizing the PICOS framework. Eligible studies comprised English-language, peer-reviewed controlled trials (either randomized or sham-controlled) with no date restrictions (database inception to November 2025), involving human subjects (regardless of age or sex; either healthy or with musculoskeletal disorders). Eligible interventions included articular manual therapy—joint mobilization (encompassing Mulligan mobilization with movement) and/or HVLA thrust manipulation—administered to spinal or peripheral joints, documented with technique, dosage, and region, and compared against sham/placebo manual therapy or no-intervention control. Research studies were mandated to present a minimum of one direct quantitative outcome related to proprioception (e.g., JPSE, cervicocephalic kinesthetic sensibility, or other validated objective metrics). The exclusion criteria encompassed non-human, in vitro, or cadaveric studies; uncontrolled studies; abstracts lacking comprehensive data; publications in languages other than English; studies focusing solely on indirect sensorimotor outcomes (such as balance, strength, or range of motion); non-articular techniques (including massage, myofascial release, Instrument-Assisted Soft Tissue Mobilization, and Muscle Energy Techniques—METs); multimodal interventions where the distinct effect of articular manual therapy could not be discerned; and studies that compared only against active interventions without an appropriate sham or no-intervention control group.

### 2.3. Information Sources and Search Strategy

A comprehensive search was performed in PubMed, Scopus, CINAHL, and MEDLINE Complete from inception to November 2025. Due to feasibility constraints and the lack of resources for translation, we limited inclusion to English-language publications. These databases were selected to provide complementary coverage: PubMed and MEDLINE Complete for biomedical literature, CINAHL for allied-health/rehabilitation research, and Scopus for broad interdisciplinary indexing and citation coverage. Database selection was also informed by institutional access and feasibility considerations, as well as the substantial overlap in indexing across major bibliographic platforms for this topic. To minimise the risk of missing eligible trials, we screened reference lists of included studies and relevant reviews and performed forward citation tracking. Search terms integrated manual therapy (e.g., mobilization, manipulation, HVLA, traction; Maitland/Mulligan) and proprioception (e.g., JPS, kinesthesia, force sense, force reproduction, threshold for detection of passive motion), applying filters for randomized or controlled experimental designs where available. The search syntax and indexing terms were adapted for each database. Full electronic search strategies for all databases (including applied limits/filters) are provided in Supplementary Table S2. Reporting of the literature searches followed PRISMA-S guidance; Supplementary Table S2 provides the full search string exactly as run for each database (including controlled vocabulary where applicable, field tags, operators, limits), the search date, and the platform/interface used. Across databases, the search yielded 483 records (Scopus *n* = 332; PubMed *n* = 79; CINAHL *n* = 42; MEDLINE Complete *n* = 30) prior to de-duplication (Figure 1).

### 2.4. Study Selection Process

Records from all databases were merged and de-duplicated (primarily by DOI and, when unavailable, by title matching) prior to screening. Two authors (SH and EP) independently assessed records in two stages (titles/abstracts, then full texts) against prespecified eligibility criteria. Disagreements were resolved by consensus, with arbitration by a third author (MS) when needed. Reasons for full-text exclusion were recorded, and study selection was reported using a PRISMA 2020 flow diagram.

### 2.5. Data Extraction

A standardized and tested extraction form was utilized. Two reviewers (SH and ICT) independently extracted data regarding the study (year, setting, design, follow-up), participant characteristics (sample size, age, sex, diagnosis/health status), intervention specifics (type of articular manual therapy—mobilization and/or HVLA manipulation—technique, region, dosage, and session frequency/duration), and eligible comparators (sham/placebo or no intervention/usual care). Quantitative proprioception results were obtained (mostly JPSE), encompassing the assessed joint/segment, measurement technique, time intervals, and numerical data (means and standard deviations (SDs) and/or change scores); effect sizes were extracted or computed when feasible. Supplementary clinical outcomes (e.g., pain, disability, balance, range of motion, strength) were documented to facilitate interpretation. Data necessary for the risk-of-bias evaluation was also gathered. Discrepancies were addressed through discussion or by a third reviewer (MS), and related authors were consulted when essential data were absent or ambiguous.

### 2.6. Risk of Bias Assessment

Risk of bias was independently assessed using the Cochrane RoB 2 tool. Five domains were evaluated, and each study received an overall judgement of low risk, some concerns, or high risk of bias [34].

### 2.7. Data Synthesis

The findings were synthesized narratively and summarized in organized tables [28]. Research was categorized by anatomical location, manual therapy method, demographic, and proprioceptive result. A priori planning for meta-analysis was conducted when a minimum of two studies exhibited sufficient comparability [35]. Random-effects models were utilized. Continuous outcomes were aggregated utilizing mean differences (MD) or standardized mean differences (SMD; Hedges’ g) [36]. Standard deviations that were absent were calculated in accordance with Cochrane guidelines [35]. Effect sizes were interpreted based on the statistic reported/derived. Standardized mean differences (Hedges’ g) were classified as trivial (<0.20), small (0.20–0.49), moderate (0.50–0.79), or large (≥0.80) following Cohen’s conventions. For studies reporting partial eta-squared (η^2^p), values of 0.01, 0.06, and 0.14 were considered small, medium, and large, respectively [37].

### 2.8. Subgroup and Sensitivity Analyses

Pre-specified subgroup analyses were performed where adequate data were accessible. Proposed subgroup analyses encompassed the type of manual therapy (thrust manipulation versus non-thrust joint mobilization), anatomical region (spinal versus peripheral joints, and specific areas when feasible), demographic (symptomatic versus healthy individuals), and timing of outcome evaluation (immediate post-intervention versus short-term follow-up).

Sensitivity analyses were conducted to assess the robustness of the findings by omitting studies deemed to have a high risk of bias and by analyzing the impact of critical analytical choices, including the use of post-intervention ratings compared to change scores. Sensitivity analyses were conducted solely when a sufficient number of papers contributed to a specific comparison.

### 2.9. Certainty of Evidence and Publication Bias

Certainty of evidence was assessed using the GRADE framework [38,39]. Publication bias was not formally assessed because fewer than 10 studies contributed to pooled analyses [35].

## 3. Results

### 3.1. Study Selection (PRISMA Flow)

Database searches identified 483 records (Scopus *n* = 332; PubMed *n* = 79; CINAHL *n* = 42; MEDLINE Complete *n* = 30). After duplicate removal (*n* = 112), 371 records were screened by title/abstract and 353 were excluded. Eighteen full-text articles were assessed for eligibility, of which 11 were excluded (ineligible intervention *n* = 6; ineligible comparator *n* = 3; ineligible outcomes *n* = 2), resulting in seven included randomized clinical trials. Duplicate records were identified primarily by DOI and, when DOI was unavailable, by title matching; overlap was most commonly observed between PubMed and Scopus, and also between PubMed and CINAHL/MEDLINE Complete. The study identification and selection process is summarised in the PRISMA flow diagram (Figure 1).

### 3.2. Characteristics of Included Studies

Seven trials [24,26,40,41,42,43,44] published between 2018 and 2025 were included, involving symptomatic and asymptomatic adults (sample sizes 26–80). Although earlier publications were identified, none met eligibility criteria (i.e., randomized or sham-controlled trials using articular manual therapy with direct, quantitative proprioceptive outcomes). Proprioception was assessed quantitatively, most commonly using joint position sense error (JPSE). The key characteristics of included studies and interventions are summarised in Table 1 and Table 2.

### 3.3. Effects of Manual Joint Mobilization and Manipulation on Proprioception

#### 3.3.1. Cervical Spine Findings

Three trials [24,26,40] evaluated cervical proprioceptive outcomes following cervical or thoracic mobilization/manipulation. In individuals with chronic mechanical neck pain, cervical thrust manipulation resulted in significant improvements in cervicocephalic kinesthetic sensibility and JPSE during cervical rotation and extension compared with sham, with large partial eta-squared effect sizes (η^2^p = 0.23–0.36) [40]. In nonspecific neck pain, cervical mobilization improved JPSE/JPS performance during left rotation compared with sham, with no between-group differences for right rotation [24]. In contrast, thoracic thrust manipulation did not produce significant between-group differences in cervical JPS compared with a no-treatment control, despite improvements in cervical range of motion [26].

#### 3.3.2. Lumbopelvic and Pelvic Manipulation Findings

In patellofemoral pain, lumbopelvic manipulation produced immediate reductions in knee JPSE at 60° [42], whereas a four-week lumbar manipulation protocol showed no between-group effect [44]. In asymptomatic participants, pelvic manipulation did not affect knee JPS [41].

#### 3.3.3. Upper Extremity

In healthy individuals, Mulligan mobilization with movement did not improve elbow JPSE compared with sham [43].

#### 3.3.4. Proprioceptive Outcome and Heterogeneity

All included studies used objective quantitative proprioceptive measures, primarily JPSE, assessed using active or passive repositioning paradigms across the cervical spine, knee, and elbow. Proprioception was frequently reported as a secondary outcome alongside pain, disability, or functional measures. Substantial clinical and methodological heterogeneity was observed across studies, including differences in populations, treated regions, manual therapy techniques, intervention dosage, and assessment protocols. This limited comparability and precluded additional quantitative pooling beyond predefined analyses.

### 3.4. Risk of Bias Within Studies

**Figure 2:**
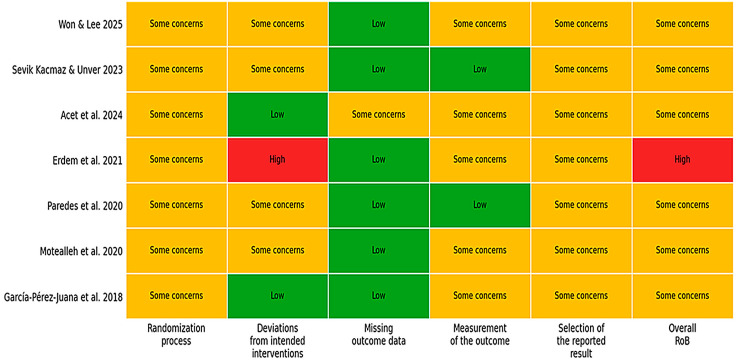
Risk of bias (RoB 2) summary for included randomized trials across domains and overall judgment (low risk, some concerns, high risk) [24,26,40,41,42,43,44].

Most trials were judged as having some concerns, primarily due to limited reporting of the randomization process (e.g., allocation concealment) and insufficient detail regarding prespecified analysis plans, raising concerns about selective reporting. Sham interventions likely reduced bias due to deviations from intended interventions; however, participant and/or assessor blinding procedures were inconsistently described. One trial using a no-intervention control was judged at high risk of bias because of increased susceptibility to performance bias [26]. Outcome measurement was generally robust because proprioceptive outcomes were assessed using standardized or instrumented methods, although assessor blinding was not consistently reported. Overall, one trial was judged at high risk of bias and the remaining trials had some concerns; no study was rated low risk overall. Across studies, the most frequent sources of concern were the randomization process and selection of the reported result (limited reporting of allocation concealment and prespecified analysis plans). In contrast, missing outcome data and outcome measurement were generally rated low risk in most trials, reflecting objective/instrumented proprioception assessment and good completeness of outcome data. A domain-level risk-of-bias summary is presented in Figure 2.

### 3.5. Meta-Analysis

**Figure 3:**
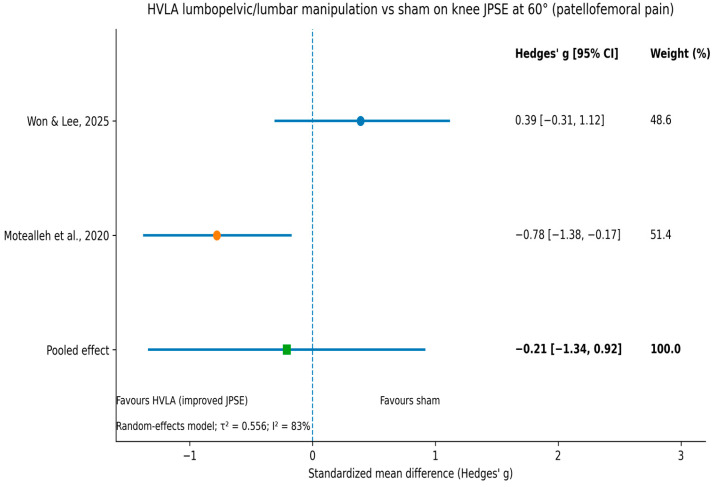
Forest plot of the random-effects meta-analysis comparing HVLA lumbopelvic/lumbar manipulation versus sham on knee joint position sense error (JPSE) at 60° of flexion in participants with patellofemoral pain (2 trials). Effect sizes are expressed as standardized mean differences (Hedges’ g) with 95% confidence intervals. Negative values indicate greater reductions in JPSE (improvement) favoring HVLA manipulation. Circles indicate individual study effects (Hedges’ g) and horizontal lines indicate 95% CIs. The square indicates the pooled random-effects estimate. The vertical dashed line represents the line of no effect (g = 0). Heterogeneity: τ^2^ = 0.556; I^2^ = 83% [42,44].

Quantitative synthesis was feasible for knee JPSE at 60° of flexion in patellofemoral pain, comparing high-velocity low-amplitude (HVLA) lumbopelvic/lumbar manipulation with sham/placebo manipulation. Two randomized controlled trials met the pooling criteria [42,44]. A random-effects model was used due to differences in intervention dosage and timing of outcome assessment. Effects were expressed as standardized mean differences (Hedges’ g) calculated from change scores (post–pre). When change-score standard deviations were not reported, they were derived from pre- and post-intervention values using conservative assumptions consistent with Cochrane guidance. The pooled effect showed no statistically significant overall benefit (Hedges’ g = −0.21, 95% CI −1.36 to 0.94) with substantial heterogeneity (I^2^ ≈ 83%). Heterogeneity reflected conflicting findings: one trial reported an immediate decrease in JPS/JPSE after treatment [42], whereas the other reported no meaningful between-group difference after a four-week intervention [44]. Sensitivity analyses using alternative assumptions for the pre–post correlation did not materially change the pooled estimate. Given the small number of studies and substantial heterogeneity, these findings should be interpreted cautiously. No further meta-analyses were undertaken because the remaining studies were insufficiently comparable in region, outcome definition, methodology, comparator, or timing. The corresponding forest plot is shown in Figure 3. Figure 3 presents the forest plot for HVLA lumbopelvic/lumbar manipulation versus sham on knee JPSE at 60°. The pooled random-effects estimate was non-significant and associated with wide confidence intervals and substantial heterogeneity (I^2^ ≈ 83%).

### 3.6. Certainty of Evidence (GRADE)

Using the GRADE framework, certainty of evidence ranged from low to moderate across outcomes/syntheses. Certainty was most commonly downgraded due to inconsistency (substantial heterogeneity across trials and outcomes) and imprecision (small sample sizes and wide confidence intervals), with additional downgrading where risk-of-bias concerns were present. For the pooled analysis of knee JPSE at 60° flexion in patellofemoral pain (two trials), certainty was rated low due to substantial heterogeneity (I^2^ ≈ 83%) and imprecision.

## 4. Discussion

### 4.1. Summary of Main Findings

This systematic review and meta-analysis synthesised evidence from randomized controlled trials examining the effects of articular manual therapy on quantitative proprioceptive outcomes. Across seven trials involving both symptomatic and asymptomatic populations, manual therapy was associated with short-term improvements in JPS or JPSE in some contexts; however, findings were heterogeneous and superiority over sham or no-intervention controls was not consistently demonstrated. The meta-analysis of knee JPSE at 60° of flexion in individuals with patellofemoral pain did not reveal a statistically significant pooled effect, highlighting substantial inconsistency between studies. Taken together, the available evidence suggests that manual therapy can influence quantitative proprioceptive outcomes (JPS/JPSE performance)**,** although the magnitude and consistency of effects vary considerably across studies. Given the marked heterogeneity in regions, techniques, comparators, and proprioception protocols, any inference across studies should be considered condition-, technique-, and outcome-specific rather than a uniform effect of manual therapy on proprioception.

### 4.2. Interpretation of Findings by Body Region and Technique

#### 4.2.1. Cervical Spine Interpretation

The cervical spine subgroup demonstrated both favorable and null findings, suggesting technique- and context-specific effects. The most robust improvements were reported after cervical HVLA thrust manipulation in chronic mechanical neck pain, with significant reductions in cervical JPSE and improvements in cervicocephalic kinesthetic sensibility versus sham and large effect sizes [40]. In contrast, evidence for non-thrust cervical mobilization was mixed: Acet et al. [24] reported direction-specific improvements (left rotation JPS) but no between-group differences for right rotation, indicating that effects may depend on the tested movement direction, baseline impairment, and/or measurement variability. Furthermore, remote thoracic HVLA manipulation did not improve cervical JPS compared with a no-treatment control despite range-of-motion gains [26], suggesting that improved mobility does not necessarily translate to improved quantitative proprioceptive performance (JPSE/JPS outcomes).

Several factors may explain these divergent findings. First, symptomatic participants with demonstrable proprioceptive deficits (e.g., chronic neck pain) likely have greater ‘room for improvement’ than asymptomatic cohorts, whereas ceiling effects may limit detectable change when baseline JPSE is near normal. Second, technique characteristics may matter: HVLA thrust may provide a brief, high-amplitude afferent ‘burst’ and short-term neuromodulatory effects, whereas mobilization may produce smaller or more gradual sensory changes. Third, comparator choice and blinding may influence observed effects; sham-controlled designs reduce contextual bias, whereas no-treatment controls may inflate apparent benefits due to expectancy and attention effects. Finally, heterogeneity in proprioception tasks (active vs. passive repositioning), tested angles, and timing (immediate vs. delayed assessment) may produce differing sensitivity to change.

#### 4.2.2. Lumbopelvic and Pelvic Manipulation Interpretation

Findings for lumbopelvic/lumbar and pelvic manipulation were conflicting. In patellofemoral pain, Motealleh et al. [42] reported immediate reductions in knee JPSE after lumbopelvic manipulation, whereas Won and Lee [44] found no meaningful between-group differences after a multi-session lumbar manipulation protocol. In asymptomatic participants, pelvic manipulation did not alter knee JPS [41]. These opposing results are consistent with the meta-analysis showing a non-significant pooled effect and substantial heterogeneity, implying that any benefit is not consistent across trials.

Potential explanations include differences in baseline status (symptomatic vs. asymptomatic), intervention dose (single-session immediate effects vs. repeated sessions), and timing of outcomes. Immediate post-treatment testing may capture transient changes in sensory thresholds or pain-related inhibition, whereas follow-up assessments may reflect whether effects persist beyond short-lived neuromodulation. Methodological differences may also contribute, including varying sham credibility, measurement error at different joint angles, and whether outcomes were primary or secondary endpoints (with secondary outcomes often underpowered).

#### 4.2.3. Peripheral Joints

Evidence for peripheral joint mobilization/manipulation was limited and largely null in the included trials. Mulligan mobilization with movement at the elbow did not improve elbow JPSE beyond sham in healthy individuals [43], and pelvic manipulation did not affect knee proprioception in asymptomatic participants [41]. These findings suggest that when clear proprioceptive deficits are not present, passive manual techniques alone may have limited capacity to produce measurable changes. Ceiling effects, short-lived sensory modulation, and the absence of task-specific practice that drives motor learning may all limit detectable improvements. Clinically, this supports using manual therapy—where appropriate—as an adjunct to enable or enhance engagement in active sensorimotor retraining rather than as a stand-alone proprioceptive intervention.

### 4.3. Comparison with Previous Systematic Reviews

Previous systematic reviews of manual therapy have primarily focused on pain, disability, and functional outcomes, with proprioceptive measures rarely included or analysed separately [45,46,47]. Conversely, reviews of proprioceptive or sensorimotor training have demonstrated improvements in proprioceptive performance and clinical outcomes but have largely centred on active exercise interventions, with manual therapy typically included only as part of multimodal programmes [48,49].

By restricting inclusion to articular manual therapy techniques and direct quantitative proprioceptive outcomes, the present review provides a more focused synthesis of the independent effects of manual therapy on proprioception. The observed heterogeneity aligns with broader manual therapy literature, which emphasises variability in neurophysiological responses and the influence of contextual and individual factors [14,15].

### 4.4. Potential Mechanisms

Articular manual therapy provides a brief mechanical stimulus that can increase afferent discharge from muscle spindles, Golgi tendon organs, joint receptors, and cutaneous mechanoreceptors, thereby altering the inflow of somatosensory information to the spinal cord and supraspinal centers [5,6,16,17,20]. Contemporary models propose that this afferent ‘barrage’ may modulate both peripheral and central processing, contributing to short-term changes in pain and sensorimotor function [18,19]. In symptomatic conditions, reduced pain and altered sensory gain may secondarily improve repositioning performance (i.e., JPS/JPSE) by reducing pain-related inhibition and improving motor output consistency [21,22].

At the spinal level, experimental and clinical work indicates that manual therapy can influence nociceptive processing and endogenous modulation (e.g., changes in conditioned pain modulation or related measures of pain facilitation/inhibition), which may create a short window where sensorimotor performance improves immediately post-intervention [21,22]. At the supraspinal level, studies suggest that spinal manipulation can transiently modify corticospinal excitability, consistent with engagement of motor-related circuitry that could plausibly affect sensorimotor integration and proprioceptive control (especially in the presence of baseline deficits) [19,20].

At a molecular and neuroimmune level, evidence—primarily from mechanistic and preclinical research—suggests that manual therapy may be associated with short-term changes in neurochemical and inflammatory signaling. For example, animal work indicates that manipulation can activate anti-inflammatory cytokine pathways (e.g., IL-10) in the spinal cord, which could theoretically reduce neuroinflammation-related sensitization and influence sensorimotor function indirectly via pain modulation [23]. However, because most mechanistic evidence relates to analgesia rather than direct proprioceptive endpoints, the most defensible interpretation is that manual therapy effects on JPS/JPSE are predominantly short-lived and state-dependent (e.g., altered pain/sensory thresholds or central gain), rather than durable proprioceptive ‘learning’ [18,19,22]. This is consistent with the heterogeneous trial findings in this review, where benefits were more apparent in some symptomatic contexts and at immediate time points, while null effects occurred when baseline deficits were minimal, comparators were rigorous (sham), or outcomes were assessed later.

Experimental studies report transient changes in corticospinal excitability after manipulation [50], and immediate modulation of pain facilitation (e.g., temporal summation) [51], while some human studies report short-term changes in circulating biomarkers related to pain/stress responses [52].

### 4.5. Certainty of Evidence and Clinical Implications

The overall certainty of evidence ranged from low to moderate, primarily limited by heterogeneity, imprecision, and small sample sizes. Although several trials demonstrated statistically significant improvements in proprioceptive outcomes following manual therapy, superiority over sham interventions was inconsistent.

Clinically, these findings suggest that manual therapy should not be considered a stand-alone intervention for improving proprioception. Instead, it may serve a complementary role within a broader rehabilitation framework, facilitating participation in targeted proprioceptive and motor control exercises, particularly in symptomatic populations with demonstrable proprioceptive deficits.

### 4.6. Limitations and Future Directions

The available evidence is limited by small sample sizes, short-term follow-up (often immediate post-intervention testing), and substantial heterogeneity in populations, treated regions, manual therapy techniques, dosage, and proprioception assessment protocols (e.g., task type, target angles, and timing), which restricted comparability and limited quantitative pooling to a single meta-analysis. Several trials reported proprioception as a secondary outcome and were unlikely to be adequately powered to detect between-group differences in JPSE/JPS outcomes. This heterogeneity also reduces confidence in identifying consistent patterns even within the narrative synthesis.

Methodological limitations within the included trials also affected certainty. Incomplete reporting of allocation concealment, blinding procedures, and prespecified analysis plans contributed to risk-of-bias concerns in multiple studies. In addition, sham credibility and the use of no-treatment controls in some comparisons may have influenced estimates via expectancy and performance effects.

At the review level, although searches were conducted from inception to November 2025 with supplementary methods (reference list screening and forward citation tracking), inclusion was restricted to English-language publications due to feasibility constraints, introducing potential language bias and limiting generalisability. We also did not search all possible databases (e.g., Embase or Web of Science) and did not conduct a formal search of grey literature or clinical trial registries; therefore, relevant unpublished or non-indexed studies may have been missed. Publication bias could not be formally assessed because fewer than 10 studies contributed to pooled analyses.

Future research should include adequately powered randomized or sham-controlled trials with standardized proprioception protocols, longer follow-up, and transparent reporting of randomization and blinding. Future research should include adequately powered randomized or sham-controlled trials with standardized proprioception protocols, longer follow-up, and transparent reporting of randomization and blinding. Methodological priorities include (i) adoption of standardized, clearly reported proprioception protocols (task type, target angles, active vs. passive repositioning, familiarization, and reliability/measurement error), (ii) prespecified primary proprioception outcomes with adequate sample-size justification, (iii) credible sham comparators with assessor blinding and transparent reporting of allocation concealment, and (iv) longer follow-up with repeated assessments to determine durability beyond immediate effects. Harmonized reporting of outcomes (means/SDs or change scores with SDs) would facilitate meta-analysis. Trials should also clarify whether any short-term changes in proprioception translate into clinically meaningful improvements in motor control, function, recurrence, or injury risk—particularly when manual therapy is combined with structured sensorimotor training. Trials should also clarify whether any short-term changes in proprioception translate into clinically meaningful improvements in motor control, function, recurrence, or injury risk—particularly when manual therapy is combined with structured sensorimotor training.

## 5. Conclusions

Across seven randomized trials (2018–2025), articular manual therapy (joint mobilization and/or HVLA thrust manipulation) may produce immediate improvements in quantitative proprioception (JPS/JPSE) in some musculoskeletal conditions; however, effects are inconsistent and are not consistently superior to sham/no-intervention controls. Meta-analysis for knee JPSE at 60° in patellofemoral pain showed no statistically significant pooled effect and substantial heterogeneity. Overall, current evidence does not support manual therapy as a stand-alone approach to improve proprioception; any benefit appears condition- and technique-specific and likely short-term.

## Figures and Tables

**Table 1:** The main characteristics of the included studies.

Study (Year)	Population	Design/Comparator	Manual Therapy Technique	Proprioceptive Outcome	Main Proprioceptive Findings	Effect Size
Won & Lee (2025) [44]	Patellofemoral pain syndrome (*n* = 30)	RCT; lumbar manipulation vs. Placebo	Lumbar spinal manipulation (HVLA)	Knee JPSΕ (30°, 45°, 60°)	NS between-group; within-group improvement at 30° and 45° (MT group).	Small *
Sevik Kacmaz & Unver (2023) [43]	Healthy adults (*n* = 56)	RCT; MWM vs. sham	Mulligan mobilization with movement (elbow)	Elbow JPSE (70°, 110°)	No significant group × time interaction; MWM not superior to sham.	Trivial
Acet et al. (2024) [24]	Nonspecific neck pain (*n* = 60)	RCT; mobilization vs. sham	Cervical joint mobilization	Cervical JPSE (left/right rotation)	Left rotation: significant improvement vs. sham; right rotation: NS between-group.	Small–moderate *
Erdem et al. (2021) [26]	Mechanical neck pain (*n* = 80)	RCT; manipulation vs. no intervention	Thoracic thrust manipulation (HVLA)	Cervical JPSE	NS between-group differences in cervical JPS/JPS error (vs no intervention).	Trivial
Paredes et al. (2020) [41]	Asymptomatic adults (*n* = 26)	RCT; global pelvic manipulation vs. sham	Global pelvic manipulation (HVLA)	Knee JPSΕ (30°, 60°)	NS between- or within-group changes in knee repositioning outcomes.	Small–moderate *
Motealleh et al. (2020) [42]	Patellofemoral pain (*n* = 44)	RCT; lumbopelvic manipulation vs. sham	Lumbopelvic manipulation (HVLA)	Knee JPSE (20°, 60°)	Immediate reduction in knee JPSE at 60° vs. sham (favours MT).	Moderate–large
García-Pérez-Juana et al. (2018) [40]	Chronic mechanical neck pain (*n* = 54)	RCT; cervical thrust vs. sham	Cervical thrust manipulation (HVLA)	Cervical JPSE (extension, rotation)	Significant group × time effects favouring MT; reductions in cervical JPSE exceeded MDC.	Moderate–large

Abbreviations: RCT, randomized controlled trial; HVLA, high-velocity, low-amplitude; MWM, mobilization with movement; JPSE, joint position sense error. * Effect size estimated from reported statistics; not all trials reported standardized effect sizes; MDC: minimal detectable change (smallest change exceeding measurement error; often reported as MDC_95 when specified).

**Table 2:** Pre- and post-intervention proprioceptive outcomes and percentage change.

Study (Year)	Body Region	Proprioceptive Outcome	Manual Therapy Group (Pre → Post)	Comparator (Pre → Post)	% Change (MT vs. Control) ^†^	Between-Group Effect
Won & Lee (2025) [44]	Knee	JPSE (°) at 30°	5.84 → 3.64	6.06 → 5.53	−37.7% vs. −8.7%	NS
		JPSE (°) at 45°	4.73 → 2.55	4.04 → 3.51	−45.8% vs. −13.1%	NS
		JPSE (°) at 60°	3.86 → 4.04	5.02 → 3.91	+4.7% vs. −22.1%	NS
Sevik Kacmaz & Unver (2023) [43]	Elbow	JPSE (°) at 70°	7.05 → 3.18	6.89 → 4.38	−54.9% vs. −36.4%	NS
		JPSE (°) at 110°	5.85 → 2.62	2.33 → 3.96	−55.2% vs. +69.9%	NS
Acet et al. (2024) [24]	Cervical	Left rotation JPSE (°)	4.15 → 1.65	4.01 → 3.74	−60.2% vs. −6.7%	Sig (favours MT)
		Right rotation JPSE (°)	4.70 → 2.11	5.31 → 4.51	−55.1% vs. −15.1%	NS
Erdem et al. (2021) [26]	Cervical	JPSE (°), multiple directions	Median Δ = 0 to −2°	Median Δ = 0°	≈−40% (extension only) *^,‡^	NS
Paredes et al. (2020) [41]	Knee	Active JPSE (°) at 30°	≈5.0 → 4.5 ^‡^	≈7.5 → 7.0 ^‡^	−10.0% vs. −6.7%	NS
		Passive JPSE (°) at 60°	≈5.5 → 4.5 ^‡^	≈3.8 → 3.5 ^‡^	−18.2% vs. −7.9%	NS
Motealleh et al. (2020) [42]	Knee	JPSE (°) at 60°	6.58 → 4.48	5.91 → 6.05	−31.9% vs. +2.4%	Sig (favours MT)
García-Pérez-Juana et al. (2018) [40]	Cervical	JPSE (°), rotation	4.1 → 3.0	5.1 → 5.5	−26.8% vs. +7.8%	Sig (favours MT)

Abbreviations: JPSE, joint position sense error; MT, manual therapy; NS, not significant between-group difference; Sig, significant between-group difference (favours MT). ^†^ Percentage changes are descriptive and not intended as effect size estimates. ^‡^ ≈ indicates values approximated from reported data (e.g., figures or medians) when exact means were unavailable. * For Erdem et al., the percent change is an approximate descriptive estimate derived from reported median changes and is direction-specific.

## Data Availability

Data extracted and analysed during the current study are available from the corresponding author upon reasonable request.

## Supplementary Materials

The following supporting information can be downloaded at: https://www.mdpi.com/article/10.3390/jfmk11010059/s1, Supplementary Table S1. Prisma checklist; Supplementary Table S2. Full electronic search strategies.

## Abbreviations

The following abbreviations are used in this manuscript:

CI	Confidence interval
CINAHL	Cumulative Index to Nursing and Allied Health Literature
GRADE	Grading of Recommendations Assessment, Development and Evaluation
HVLA	High-velocity, low-amplitude (thrust)
I^2^	I-squared (heterogeneity statistic)
JPSE	Joint position sense error
JPS	Joint position sense
MD	Mean difference
MEDLINE	Medical Literature Analysis and Retrieval System Online
MWM	Mobilization with movement
PICOS	Population, Intervention, Comparator, Outcomes, Study design
PRISMA	Preferred Reporting Items for Systematic Reviews and Meta-Analyses
PROSPERO	International Prospective Register of Systematic Reviews
RCT	Randomized controlled trial
RoB 2	Risk of Bias 2 (Cochrane tool)
SD	Standard deviation
SMD	Standardized mean difference
