# Supplementary material for: The Effect of Joint Mobilization and Manipulation on Proprioception: Systematic Review with Limited Meta-Analysis
Source: J Funct Morphol Kinesiol. 2026 Jan 29;11(1):59. doi: 10.3390/jfmk11010059 (PMC12921973; doi:10.3390/jfmk11010059)
Supplement: Supplementary file 1 [file jfmk-11-00059-s001.zip › jfmk-4091601-Supplementary Table S2.pdf]

Supplementary Table S2. Full electronic search strategies

Databases searched: PubMed, Scopus, CINAHL (EBSCOhost), MEDLINE Complete (EBSCOhost)

Search date: November 2025

Coverage: from inception to November 2025 (no date limits)

Limits/filters applied (where available): Humans; English; Clinical trial, Randomized controlled trial, Controlled clinical trial, Evaluation study, Comparative study

Records identified from databases: 483 (Scopus 332; PubMed 79; CINAHL 42; MEDLINE Complete 30)

Duplicates removed before screening: 112 (identified by DOI; if DOI unavailable, by title matching)

Database	Platform/interface	Search query (exact as run)	Limits/filters applied	Records retrieved (n)
PubMed	PubMed (NCBI)	(manual therapy[Title/Abstract] OR mobilization[Title/Abstract] OR mobilisation[Title/Abstract] OR manipulation[Title/Abstract] OR "spinal manipulation"[Title/Abstract] OR "joint mobilization"[Title/Abstract] OR "joint mobilisation"[Title/Abstract] OR "high-velocity low-amplitude"[Title/Abstract] OR HVLA[Title/Abstract] OR traction[Title/Abstract] OR "joint distraction"[Title/Abstract] OR Maitland[Title/Abstract] OR Mulligan[Title/Abstract]) AND (proprioception[Title/Abstract] OR proprioceptive[Title/Abstract] OR "joint position sense"[Title/Abstract] OR "joint position error"[Title/Abstract] OR "position sense"[Title/Abstract] OR kinesthesia[Title/Abstract] OR kinaesthesia[Title/Abstract] OR "cervicocephalic kinesthetic sensibility"[Title/Abstract]	Species: Humans<br>Language: English<br>Article types: Clinical Trial; Randomized Controlled Trial; Controlled Clinical Trial; Evaluation Study; Comparative Study<br>Date limits: None (from inception to November 2025)	79

		OR reposition*[Title/Abstract] OR "force sense"[Title/Abstract] OR "sense of force"[Title/Abstract] OR "sense of effort"[Title/Abstract] OR "force reproduction"[Title/Abstract] OR TTDPM[Title/Abstract] OR "threshold for detection of passive motion"[Title/Abstract] OR "threshold to detect passive motion"[Title/Abstract])		
Scopus	Scopus (Elsevier)	TITLE-ABS-KEY ("manual therapy" OR mobilization OR mobilisation OR manipulation OR "spinal manipulation" OR "joint mobilization" OR "joint mobilisation" OR "high-velocity low-amplitude" OR HVLA OR traction OR "joint distraction" OR Maitland OR Mulligan) AND TITLE-ABS-KEY (proprioception OR proprioceptive OR "joint position sense" OR "joint position error" OR "position sense" OR kinesthesia OR kinaesthesia OR "cervicocephalic kinesthetic sensibility" OR reposition* OR "force sense" OR "sense of force" OR "sense of effort" OR "force reproduction" OR TTDPM OR "threshold for detection of passive motion" OR "threshold to detect passive motion")	Language: English Document type: Article Date limits: None (from inception to November 2025) Humans / study design limits: not directly available as database filters; applied during screening	332
CINAHL	CINAHL via EBSCOhost	((TI ("manual therapy" OR mobilization OR mobilisation OR manipulation OR "spinal manipulation" OR "joint mobilization" OR "joint mobilisation" OR "high-velocity low-amplitude" OR HVLA OR traction OR "joint distraction" OR Maitland OR Mulligan)) OR (AB ("manual therapy" OR mobilization OR mobilisation OR manipulation OR "spinal manipulation" OR "joint mobilization" OR "joint mobilisation" OR "high-velocity low-amplitude" OR HVLA OR traction OR "joint distraction" OR Maitland OR Mulligan))) AND ((TI (proprioception OR proprioceptive OR "joint position sense" OR "joint	Language: English Humans Publication types / study designs: Clinical trial, RCT, Controlled clinical trial, Evaluation study, Comparative study (applied using available EBSCO filters) Date limits: None (from inception to	42

		position error" OR "position sense" OR kinesthesia OR kinaesthesia OR "cervicocephalic kinesthetic sensibility" OR reposition* OR "force sense" OR "sense of force" OR "sense of effort" OR "force reproduction" OR TTDPM OR "threshold for detection of passive motion" OR "threshold to detect passive motion")) OR (AB (proprioception OR proprioceptive OR "joint position sense" OR "joint position error" OR "position sense" OR kinesthesia OR kinaesthesia OR "cervicocephalic kinesthetic sensibility" OR reposition* OR "force sense" OR "sense of force" OR "sense of effort" OR "force reproduction" OR TTDPM OR "threshold for detection of passive motion" OR "threshold to detect passive motion")))	November 2025)	
MEDLINE Complete	MEDLINE Complete via EBSCOhost	((TI ("manual therapy" OR mobilization OR mobilisation OR manipulation OR "spinal manipulation" OR "joint mobilization" OR "joint mobilisation" OR "high-velocity low-amplitude" OR HVLA OR traction OR "joint distraction" OR Maitland OR Mulligan)) OR (AB ("manual therapy" OR mobilization OR mobilisation OR manipulation OR "spinal manipulation" OR "joint mobilization" OR "joint mobilisation" OR "high-velocity low-amplitude" OR HVLA OR traction OR "joint distraction" OR Maitland OR Mulligan))) AND ((TI (proprioception OR proprioceptive OR "joint position sense" OR "joint position error" OR "position sense" OR kinesthesia OR kinaesthesia OR "cervicocephalic kinesthetic sensibility" OR reposition* OR "force sense" OR "sense of force" OR "sense of effort" OR "force reproduction" OR TTDPM OR "threshold for detection of passive motion" OR "threshold to detect passive motion")) OR (AB (proprioception OR proprioceptive OR "joint	Language: English<br>Humans<br>Publication types / study designs: Clinical trial, RCT, Controlled clinical trial, Evaluation study, Comparative study (applied using available EBSCO filters)<br>Date limits: None (from inception to November 2025)	30

		position sense" OR "joint position error" OR "position sense" OR kinesthesia OR kinaesthesia OR "cervicocephalic kinesthetic sensibility" OR reposition* OR "force sense" OR "sense of force" OR "sense of effort" OR "force reproduction" OR TTDPM OR "threshold for detection of passive motion" OR "threshold to detect passive motion")))		

Note: The search strategy was adapted for each database syntax; equivalent terms and limits were applied where available. Reporting of the literature searches followed PRISMA-S guidance.
